# *Mycobacterium abscessus* infection in a young man with cystic fibrosis: a case report and literature review

**DOI:** 10.3389/fped.2026.1737211

**Published:** 2026-05-26

**Authors:** Ling Luo, Zhuoyao Guo, Weicheng Chen, Yanghong Zheng, Chen Chen, Qiang Li, Na Wang, Yingqun Ji, Jing Hua

**Affiliations:** 1 Department of Pulmonary and Critical Care Medicine, Shanghai East Hospital Affiliated to Tongji University, Tongji University School of Medicine, Shanghai, China; 2 Department of Respirology, Children's Hospital of Fudan University, Shanghai, China; 3 Department of Cardiothoracic Surgery, Children's Hospital of Fudan University, Shanghai, China

**Keywords:** bronchiectasis, case report, CFTR gene, CFTR modulators, cystic fibrosis, *Mycobacterium abscessus*, nontuberculous mycobacteria

## Abstract

**Background:**

Cystic fibrosis (CF) is a rare autosomal recessive disorder caused by mutations in the cystic fibrosis transmembrane conductance regulator (CFTR) gene. Although relatively common in Caucasian populations, CF is rare in China, where it frequently presents with non-specific respiratory symptoms, leading to delayed diagnosis and frequent coinfections with multidrug-resistant pathogens.

**Case report:**

A 21-year-old man presented with a 6-year history of recurrent productive cough and intermittent fever over the past 6 months. Imaging revealed bronchiectasis with evidence of infection. Metagenomic next-generation sequencing of bronchoalveolar lavage fluid identified *Staphylococcus aureus* and *Mycobacterium abscessus*. Further investigations revealed pancreatic lipomatosis, congenital absence of seminal vesicles, and fat-soluble vitamin deficiencies. CF diagnosis was confirmed by elevated sweat chloride concentration (88 mmol/L) and biallelic CFTR mutations. Clinical stability was achieved through a quadruple antimycobacterial regimen (linezolid, moxifloxacin, azithromycin, and minocycline) combined with systemic supportive care. CFTR modulator therapy was deferred due to limited access and financial constraints.

**Conclusion:**

We report a case of CF in a Chinese patient presenting with nontuberculous mycobacterial infection, a condition rarely documented in East Asian populations. We provide a review of the relevant literature, aiming to emphasize the importance of early recognition of CF, personalized antimicrobial strategies, and improved access to essential medications.

## 1 Case presentation

### 1.1 History of present illness

A 21-year-old man presented with a 6-year history of recurrent cough and purulent sputum, accompanied by intermittent fever over the preceding 6 months. Initial evaluation 6 years earlier had revealed bronchiectasis with infection on chest computed tomography (CT) and *Staphylococcus aureus* on sputum culture, treated with a 3-week antibiotic course (details unknown).Over the subsequent 6 years, the patient experienced intermittent exacerbations. Six months prior to the current admission, he developed worsening symptoms with copious yellow sputum and high fever (39.5°C). Chest CT demonstrated multilobar bronchiectasis with infection, while next-generation sequencing (NGS) of bronchoalveolar lavage fluid (BALF) obtained via bronchoscopy identified *S. aureus* and *Mycobacterium abscessus* (MAB) (Table 1). Initial anti-nontuberculous mycobacterial (NTM) therapy included intravenous tigecycline (50 mg every 12 h), imipenem-cilastatin (1 g every 12 h), amikacin (800 mg once daily, weight-adjusted), and oral azithromycin (500 mg once daily) for 40 days followed by a maintenance regimen of oral linezolid (600 mg once daily), moxifloxacin (400 mg once daily), azithromycin (500 mg twice daily), and minocycline (100 mg twice daily). A follow-up chest CT obtained 4 months later revealed progression of left lower lobe lesions. BALF cultures grew *Candida albicans* and *Aspergillus fumigatus*, prompting initiation of voriconazole (200 mg twice daily). Subsequent metagenomic NGS of BALF identified the concurrent presence of *Mycobacterium intracellulare* and *M. abscessus*. Phenotypic antimicrobial susceptibility testing was performed on the *M. abscessus* isolate, which revealed susceptibility to macrolides and aminoglycosides. Based on these findings, the anti-NTM therapy was adjusted to the following regimen: linezolid 600 mg once daily, moxifloxacin 400 mg once daily, azithromycin 500 mg once daily, and minocycline 100 mg twice daily. Notably, the patient's medical history included a duodenal ulcer and hypokalemia that was previously misdiagnosed as Bartter syndrome.

**Table 1:** Laboratory, microbiological, and genetic findings of the patient.

Category	Items	Results		Units/details	Reference range
		Preadmission	Current admission		
CBC	WBC	5.42	4.80	×10^9^/L	3.5–9.5
	Neutrophil	4.20	3.02	×10^9^/L	1.8–6.3
	Lymphocyte	1.30	0.01	×10^9^/L	0.8–4.0
	Monocyte	0.60	1.24	×10^9^/L	0.12–0.8
	RBC	4.54	4.12	×10^12^/L	4.3–5.8
	Hb	132	134.0	g/L	130–175
	PLT	319	244	×10^9^/L	125–350
Coagulation function	PT	–	12.8	seconds	11–14
	APTT	–	32.2	seconds	25–35
	D-Dimer	–	0.450	mg/L FEU	<0.5
Inflammatory markers	CRP	–	20.39	mg/L	<10
	SAA	–	127.81	mg/L	<10
	IL-6	–	25.630	pg/mL	<7
	PCT	0.106	0.339	ng/mL	<0.1
Basic metabolic panel	Sodium	–	135.47	mmol/L	137–147
	Potassium	–	4.00	mmol/L	3.5–5.3
	Calcium	2.26	2.17	mmol/L	2.1–2.6
	Creatinine	99.40	76.30	μmol/L	59–104
	ALT	43.1	18.00	U/L	<40
	AST	55.7	30.40	U/L	<40
Blood gas analysis	pH	–	7.436	–	7.35–7.45
	PaO_2_	–	80.5	mmHg	80–100
	PaCO_2_	–	31.8	mmHg	35–45
Vitamins and hormones	25-Hydroxy Vitamin D	–	3.01	ng/mL	>20
	Vitamin C	–	33	μmol/L	50–150
	Testosterone	–	1.320	ng/mL	2.8–8.8
Microbe NGS of BALF	First BALF				
	*Staphylococcus aureus*	55,702 reads		–	–
	*Mycobacterium abscessus*	64,352 reads		–	–
	Second BALF				
	MAC	Detected		–	–
	MAB	Detected		–	–
Genetic test results	Variant 1	c.2909G>A (p.G970D)			
	Variant type	Missense			
	Genomic position (hg19)	chr7:117246728			
	Exon	18			
	ACMG classification	Pathogenic (PVS1, PM2, PM3)			
	Protein effect	Gly970Asp (conserved residue)			
	Variant 2	c.3987_3988delAC (p.Q1330Vfs*6)			
	Variant type	Frameshift deletion			
	Genomic position (hg19)	chr7:117304765-117304766			
	Exon	25			
	ACMG classification	Pathogenic (PVS1, PM2)			
	Protein effect	Gln1330ValfsTer6 (premature termination)			

CBC, complete blood count; WBC, white blood cell count; RBC, red blood cell count; Hb, hemoglobin; PLT, platelet count; PT, prothrombin time; APTT, activated partial thromboplastin time; CRP, C-reactive protein; SAA, serum amyloid A; IL-6, interleukin-6; PCT, procalcitonin; ALT, alanine aminotransferase; AST, aspartate aminotransferase; NGS, next-generation sequencing; BALF, bronchoalveolar lavage fluid; MAC, *Mycobacterium avium* complex; MAB, *Mycobacterium abscessus*.

### 1.2 Diagnostic evaluation

**Figure 1:**
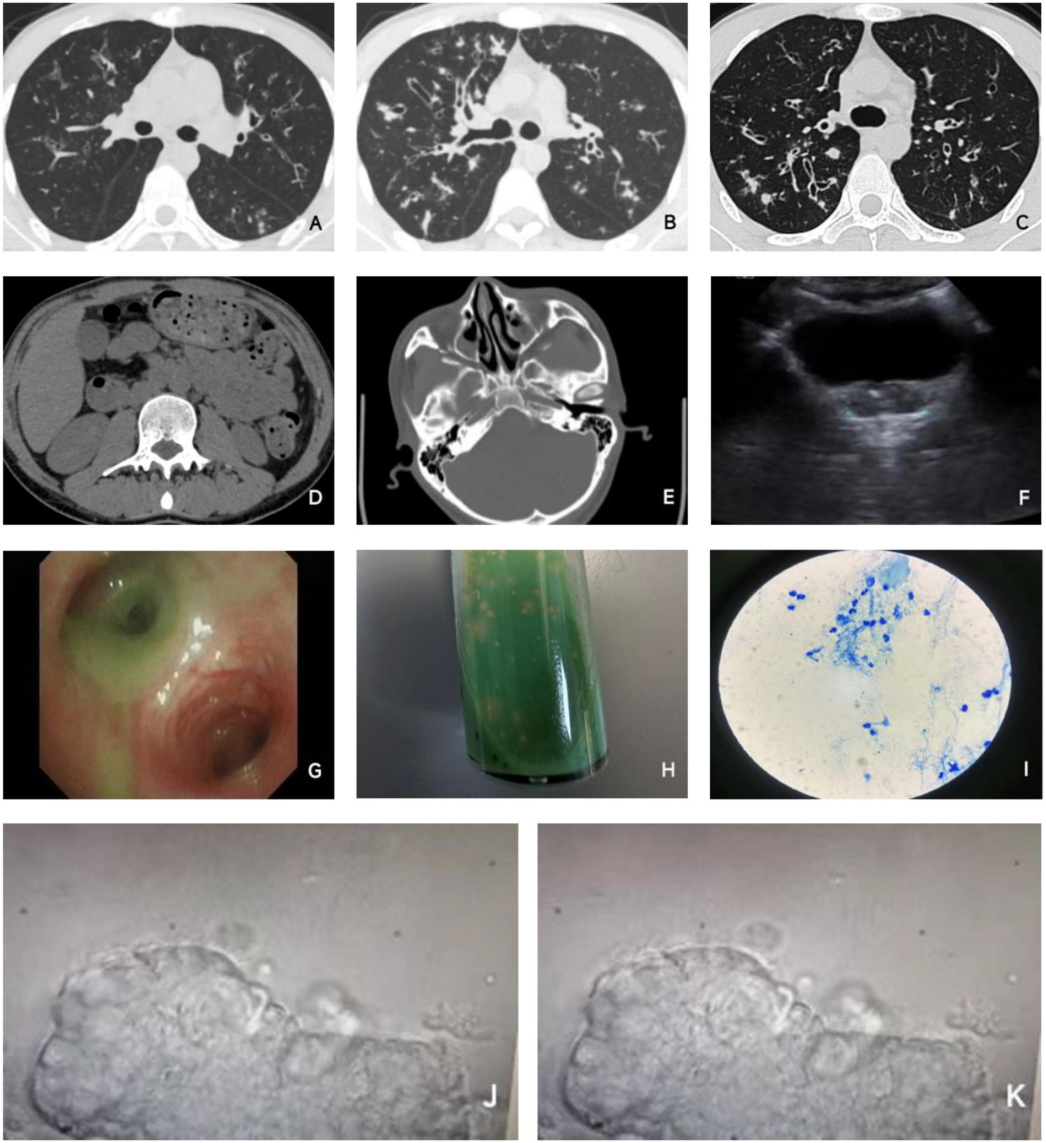
Imaging and other examinations of a 21-year-old man with cystic fibrosis. **(**A–C**)** Serial chest CT scans showing progressive bilateral bronchiectasis with peripheral patchy and nodular infiltrates. **(**D**)** Abdominal CT showing pancreatic steatosis. **(**E**)** Paranasal sinus CT showing bilateral sinusitis, mild hypertrophy of the inferior turbinates, and deviated nasal septum. **(**F**)** Genitourinary ultrasound showing congenital absence of the bilateral seminal vesicles. **(**G**)** Cryobiopsy specimen showing chronic mucosal inflammation. **(**H,I**)** BALF smear showing acid-fast bacilli (3+) and culture positive for *Mycobacterium abscessus*. **(**J,K**)** High-speed video-microscopy analysis demonstrating normal ciliary beat frequency and pattern.

Physical examination revealed a right lower pleural friction rub. Imaging findings included bronchiectasis predominantly seen in the bilateral lower lobes, pancreatic lipomatosis, bilateral sinusitis, and bilateral seminal vesicle agenesis (genitourinary ultrasound) (Figure 1). Laboratory tests revealed hypovitaminosis (25-OH vitamin D 3.01 ng/mL), exocrine pancreatic insufficiency (lipase 7.5 U/L, pancreatic amylase 5 U/L), and hypogonadism [testosterone 1.320 ng/mL, sex hormone-binding globulin 13.2 nmol/L]. BALF sputum cultures confirmed MAB, with histopathology showing chronic mucosal inflammation. High-speed video-microscopy analysis of nasal epithelial ciliary motion demonstrated normal ciliary beat frequency and pattern. Sweat chloride conductivity was 88 mmol/L (diagnostic cutoff ≥60 mmol/L), and nasal nitric oxide was 110 nL/min. Genetic testing identified biallelic pathogenic variants in the cystic fibrosis transmembrane conductance regulator (CFTR) gene (p.G970D and p.Q1330Vfs*6) (Table 1).

These findings fulfilled the diagnostic criteria for cystic fibrosis according to the Cystic Fibrosis Foundation consensus guidelines (1) and the Chinese Experts Cystic Fibrosis Consensus statement (2023) (2).

## 2 Management and outcome

A definitive diagnosis of cystic fibrosis (CF) with MAB and suspected *A. fumigatus* coinfection was established based on the clinical manifestations, medical history, physical examination, and comprehensive investigations.

Treatment strategy included linezolid 600 mg once daily, moxifloxacin 400 mg once daily, azithromycin 500 mg once daily, and minocycline 100 mg twice daily for anti-NTM therapy. Hypertonic saline and tobramycin nebulization were also prescribed. As for pancreatic enzyme replacement, pancrelipase capsules (150 mg three times daily with meals) were prescribed. In addition, vitamin D3, Calcitriol, hydration, and dietary supplementation were established. Due to limited healthcare insurance coverage in China and constrained family financial circumstances, CFTR modulator therapy was deferred. The patient and his parents opted for regular follow-up care.

## 3 Discussion and literature review

CF is an autosomal recessive disorder caused by *CFTR* gene mutations, resulting in defective chloride channel function leading to multiorgan dysfunction, predominantly affecting the respiratory, digestive, and reproductive systems. Despite its historical association with Caucasian populations, CF was officially included in China's 2018 Rare Disease Catalog, with an estimated carrier frequency of 1/167–190 and a disease prevalence of approximately 1/120,000 (3, 4). We described a 21-year-old man with CF presenting with the classic clinical triad of bronchiectasis, exocrine pancreatic insufficiency, and congenital bilateral absence of the seminal vesicles. Biallelic *CFTR* mutations (p.G970D and p.Q1330Vfs*6) were identified, consistent with the mutation spectrum observed in Chinese patients with CF. Unlike Western populations, where the p.F508del variant predominates (>80%), Chinese patients exhibit a distinct and highly heterogeneous mutation profile (5). Biallelic mutations in this population correlate with a higher prevalence of early-onset pancreatic insufficiency (68% vs. 85%–90% in Western cohorts) but with a comparatively slower rate of pulmonary function decline (1.2% vs. 2.5% annual forced expiratory volume in the first second loss). These phenotypic differences underscore the critical need for population-specific genetic screening strategies and personalized clinical management in the care of Chinese patients with CF.

CFTR dysfunction results in the production of abnormally viscous mucus that impairs mucociliary clearance, thereby creating a permissive niche for chronic microbial colonization. This process initiates a cycle of persistent airway inflammation and progressive structural lung damage, ultimately culminating in respiratory failure—the leading cause of mortality in CF. The microbiological landscape of CF lung disease in Chinese patients appears to differ from that in Western populations. While *Pseudomonas aeruginosa* remains a common pathogen globally, Chinese cohorts demonstrate a higher relative frequency of *Burkholderia cepacia* complex and NTM. In contrast, methicillin-sensitive *Staphylococcus aureus* and *Stenotrophomonas maltophilia* are more prevalent in Western registries. Notably, NTM have emerged as significant opportunistic pathogens in CF, with reported prevalence rates reaching up to 14% (6). Among these, MAB complex is particularly problematic due to its rapid growth kinetics, propensity for biofilm formation, and intrinsic resistance to multiple antibiotic classes. In the present case, the patient's respiratory history began with *S. aureus* infection during childhood and was subsequently complicated by MAB isolation. Although bronchiectasis may predispose to secondary NTM colonization, accumulating evidence suggests that *M. abscessus* and *Mycobacterium avium* complex infections are themselves potent drivers of bronchiectasis progression. Furthermore, epidemiological studies have documented the potential for person-to-person transmission of MAB within CF care centers (7), and chronic MAB infection is independently associated with an accelerated rate of lung function decline (8). Of note, ciliary motion analysis in this patient demonstrated normal beat frequency and pattern, which effectively ruled out primary ciliary dyskinesia. However, it should be acknowledged that this *ex vivo* assessment may not fully recapitulate the secondary ciliary dysfunction caused by the highly viscous mucus characteristic of CF airways *in vivo*. These findings underscore the imperative for stringent infection control measures and early pathogen surveillance in this vulnerable population.

The management of MAB infection in patients with CF remains an arduous clinical challenge due to the organism's intrinsic multidrug resistance and the limited armamentarium of effective, well-tolerated antimicrobial agents. Current international consensus guidelines recommend an intensive phase of therapy comprising a macrolide (if susceptible) combined with intravenous agents such as amikacin and a beta-lactam (e.g., imipenem or cefoxitin), followed by a prolonged continuation phase with oral and inhaled antibiotics. However, treatment outcomes are frequently suboptimal, with therapeutic success often hampered by poor drug tolerability, significant toxicity—particularly aminoglycoside-induced ototoxicity and nephrotoxicity—and the emergence of acquired drug resistance. In the present case, a personalized multidrug regimen (linezolid, moxifloxacin, azithromycin, and minocycline) was implemented based on phenotypic antimicrobial susceptibility testing and serial assessments of clinical response. The patient achieved relative clinical and radiological stability on this maintenance regimen, demonstrating that individualized therapeutic strategies—tailored to both *in vitro* susceptibility data and patient-specific tolerance—are essential for managing CF-associated NTM disease.

Comprehensive management of CF requires an integrated, multidisciplinary approach encompassing aggressive airway clearance techniques, mucolytic therapy, inhaled antibiotics, and systemic nutritional and pancreatic enzyme replacement support. While the advent of highly effective CFTR modulator therapies has fundamentally transformed the treatment paradigm and long-term prognosis for individuals with CF in high-income countries, their implementation in China remains severely constrained by prohibitive costs and the absence of national reimbursement policies. In the present case, the patient and his family elected to defer modulator therapy due to financial limitations, highlighting a critical gap in global health equity. Moreover, as survival in the CF population continues to improve, the clinical landscape is increasingly shaped by challenging pathogens such as MAB. However, it is noteworthy that MAB infection is no longer considered an absolute contraindication to lung transplantation, as several specialized centers have reported successful post-transplant outcomes following meticulous perioperative antimicrobial management (9). This case serves to emphasize the pressing need to advance CF care in China through heightened clinician awareness of disease heterogeneity, the development of standardized protocols for early NTM detection and management, and the establishment of collaborative clinical networks to elevate diagnostic precision and therapeutic outcomes nationwide.

## Data Availability

The raw data supporting the conclusions of this article will be made available by the authors, without undue reservation.
